# Peptidomimetic inhibitor of L-plastin reduces osteoclastic bone resorption in aging female mice

**DOI:** 10.1038/s41413-020-00135-9

**Published:** 2021-04-09

**Authors:** Hanan Aljohani, Joseph P. Stains, Sunipa Majumdar, Deepa Srinivasan, Linda Senbanjo, Meenakshi A. Chellaiah

**Affiliations:** 1 Department of Oncology and Diagnostic Sciences, School of Dentistry, University of Maryland, Baltimore, MD USA; 2 Department of Oral Medicine and Diagnostics Sciences, King Saud University, School of Dentistry, Riyadh, Kingdom of Saudi Arabia; 3 Department of Orthopedics, University of Maryland School of Medicine, Baltimore, MD USA

**Keywords:** Bone quality and biomechanics, Bone

## Abstract

L-plastin (LPL) was identified as a potential regulator of the actin-bundling process involved in forming nascent sealing zones (NSZs), which are precursor zones for mature sealing zones. TAT-fused cell-penetrating small molecular weight LPL peptide (TAT- MARGSVSDEE, denoted as an inhibitory LPL peptide) attenuated the formation of NSZs and impaired bone resorption in vitro in osteoclasts. Also, the genetic deletion of LPL in mice demonstrated decreased eroded perimeters and increased trabecular bone density. In the present study, we hypothesized that targeting LPL with the inhibitory LPL peptide in vivo could reduce osteoclast function and increase bone density in a mice model of low bone mass. We injected aging C57BL/6 female mice (36 weeks old) subcutaneously with the inhibitory and scrambled peptides of LPL for 14 weeks. Micro-CT and histomorphometry analyses demonstrated an increase in trabecular bone density of femoral and tibial bones with no change in cortical thickness in mice injected with the inhibitory LPL peptide. A reduction in the serum levels of CTX-1 peptide suggests that the increase in bone density is associated with a decrease in osteoclast function. No changes in bone formation rate and mineral apposition rate, and the serum levels of P1NP indicate that the inhibitory LPL peptide does not affect osteoblast function. Our study shows that the inhibitory LPL peptide can block osteoclast function without impairing the function of osteoblasts. LPL peptide could be developed as a prospective therapeutic agent to treat osteoporosis.

## Introduction

Osteoporosis is a systemic skeletal disease characterized by decreased bone formation by osteoblasts with increased osteoclastic bone resorption.^1^ Unlike postmenopausal osteoporosis, which occurs due to a sudden withdrawal of circulating estrogens leading to markedly increased osteoclast bone resorption, aging affects both osteoclast and osteoblast lineages. Aging-related bone loss was associated with increased osteoclastogenesis^2^ and altered the conditions that may facilitate the differentiation of mesenchymal stem cells into adipocytes and possibly reduce osteoblast differentiation.^3,4^ Therefore, novel and improved therapies are critically needed to target osteoclast activity more efficiently without affecting osteoblasts’ function.

Plastins (L-, T- and I-plastin) are a family of tissue-specific actin-binding proteins. Although L- and T-plastin have a regulatory role in cytoskeletal reorganization, only L-plastin (LPL) can efficiently bundle the actin filaments.^5,6^ Phosphorylation of LPL on Ser-5 and Ser-7 regulates the actin-bundling processes essential for cytoskeletal rearrangements.^7–9^ Osteoclasts require actin-bundling to form the sealing ring structure that is obligated to resorb bone. Previously, we demonstrated in osteoclasts that LPL regulates the formation of actin aggregates at the early phase of sealing ring formation, denoted as nascent sealing zones (NSZs). In the NSZs, these actin aggregates mature into fully functional sealing rings by αvβ3 signaling in resorbing osteoclasts.^10^

We have recently confirmed that LPL phosphorylation on serine 5 and −7 residues increase F-actin’s bundling capacity and the formation NSZs. For this, we transduced osteoclasts with TAT-fused full length (FL-LPL) and mutated FL-LPL (Ser-5 and Ser-7 to Ala-5 and Ala-7). An increase in the number of sealing rings was observed with FL-LPL and not with the mutated FL-LPL protein (11). Next, we transduced osteoclasts with TAT-fused small molecular weight amino-terminal LPL peptide (sNT-LPL) containing serine 5 and 7 aa residues (TAT-1MARGSVSDEE10). TAT-fused scrambled peptide (TAT-SRSGMVEEAD) was used as a control. We showed that the transduction of TAT-1MARGSVSDEE10 (*hereafter denoted as an inhibitory LPL peptide*) significantly decreased the phosphorylation of endogenous LPL. Therefore, this diminished the actin-bundling process involved in the formation of NSZs and hence mature sealing rings. These osteoclasts were less resorptive in vitro when plated on the dentine matrix.^11,12^ However, the inhibitory LPL peptide did not affect bone formation by osteoblasts in vitro.^13^ Likewise, osteoclasts from LPL knockout mice (LPL^-/-^) failed to demonstrate either NSZs or mature sealing rings and are defective in resorption function in vitro.^14^ In vivo LPL^-/-^ mice have an osteopetrotic phenotype, consistent with a clear role of LPL in osteoclast function.^9,14^ Notably, no apparent effect of LPL deletion on osteoblast function was detected.^14^

Taken together, LPL^-/-^ mice are osteopetrotic^14^ and that TAT-fused LPL peptide can enter into osteoclasts, competitively inhibit LPL phosphorylation, and block NSZ formation in vitro.^11,12^ Thus, we sought to examine the efficacy of the inhibitory LPL peptide in vivo. In this paper, our principal goal was to identify whether injection of the inhibitory LPL peptide into aging mice will suppress the function of cellular LPL in the formation of NSZs and increase bone density by targeting osteoclasts while preserving osteoblast activity. We have investigated the in vivo effects of LPL peptides (inhibitory and scrambled) in aging C57/BL6 female mice. Subcutaneous injection (~1.5 mg·kg^−1^ body weight) was given in 36 week old mice for 14 weeks., and then a systematic evaluation of the skeletal phenotype was performed. Our data indicate that the inhibition of LPL function in osteoclasts may provide a new strategy to prevent age-related bone loss without affecting the function of osteoblasts.

## Results

### Analysis of the effect of transduced LPL peptide on actin modulation and dentine resorption by osteoclasts in vitro

**Fig. 1:**
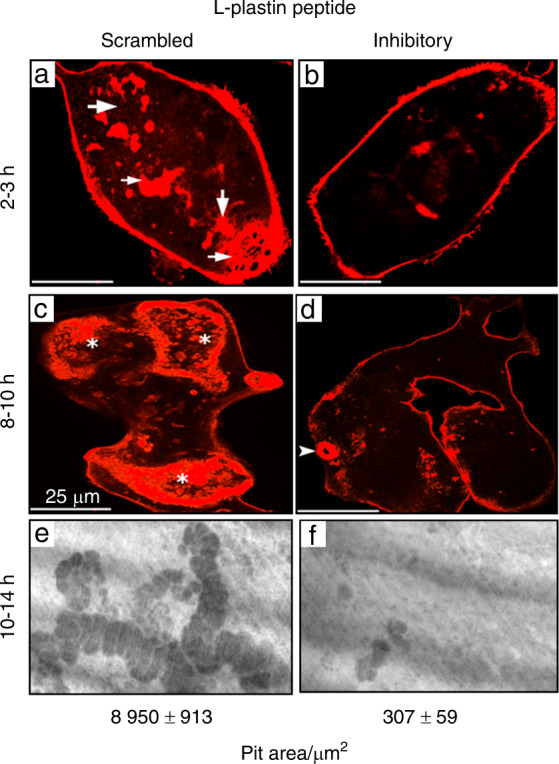
Osteoclasts were stained for actin with rhodamine-phalloidin, and confocal images of osteoclasts are shown (**a**–**d**). An arrowhead points to a small sealing ring in (**d**). Scale Bar-25 µm. **e** and **f** Dentine resorption assay in vitro: Osteoclasts plated on dentine matrix were incubated with scrambled (**e**) or inhibitory (**f**) LPL peptide in the presence of TNF-α for 10–14 h. Resorption pits were photographed under a 40X objective of phase-contrast microscopy (magnification is ×400). Resorbed areas of approximately ~25–30 pits were quantified from three slices per treatment/experiment (*n* = 3) and averaged over two different experiments. Statistical analysis of the pit area was performed by Student’s test and provided at the bottom. ***P < 0.001 vs. scrambled peptide transduced osteoclasts (mean ± SEM)

To validate the effectiveness of the inhibitory peptide (1MARG**S**V**S**DEE10) of LPL, we confirmed the effect of this peptide in osteoclasts derived from RAW cells in vitro (Fig. 1). Osteoclasts transduced with either inhibitory or scrambled LPL peptide for 15 min were plated on dentine slices in the presence of respective peptides and TNF-α for 2–3 h (Fig. 1a, b) or 8–10 h (c and d). Subsequently, cells were stained for actin with rhodamine-phalloidin. A considerable decrease in the number of NSZs and sealing rings were observed in osteoclasts transduced with the inhibitory LPL peptide (Fig. 1, panels b and d) as compared with the ones transduced with scrambled peptide (a and c). Pit assay was used to measure the resorption activity and is expressed as the resorption area at the bottom of the figure. Inhibition of sealing ring formation by the inhibitory LPL peptide affected the resorbing activity of the osteoclasts plated on the dentine matrix (panel f). Sealing ring formation in osteoclasts transduced with scrambled peptide corresponds (Fig. 1, panel c) with an increase in pit areas (Fig. 1, panel e). Together, these observations validate the role of LPL in the regulation of sealing ring formation and resorption function in osteoclasts.

### Effect of the inhibitory LPL peptide on the microarchitecture of bones isolated from mice injected with peptides of interest

Here, we used the aging mouse model to identify LPL as a potential therapeutic target to control the bone loss induced by aging. C57BL/6 mice are recognized as an attractive mice model for aging-related studies.^15^ Female C57BL/6 mice at 36 weeks (9 months old) of age were given injections for 14 weeks, as described in the Methods sections. Mice were 50 weeks (~12.5 months) of age at the time of sacrifice. While only a model for early aging, C57BL/6 mice demonstrate a considerable decrease in bone mass by 12 months of age. Trabecular bone loss begins at about 3 months in C57BL/6 mice and disappears by 8–12 months in male and female mice. However, the extent of bone loss is more in female mice than males.^16,17^

### General observations in mice during the injection period of 14 weeks

As indicated in the Methods section, the injection was given every day (5 days per week) for 7 weeks at the initial phase of the experiments. Initially, the mice looked healthy and active. However, after week 5, a few of the mice slowly started losing their hairs and appeared lethargic. After week 7, mice were less active and showed more hair loss. Therefore, the injections were given on alternate days (3 days per week) for another 7 weeks. From week 9 onwards, all mice looked had recovered, appeared active, and healthy. Animal weights were taken every 2 weeks, and pictures were taken during the 13th week before sacrificing them on the 14th week. All mice looked normal and healthier with well-grown hair, but they were generally slower, which may be due to aging (Fig. S1A). No significant alteration in the body weight was observed between the two groups tested for 13 weeks (Fig. S1B).

### Histological analyses of soft organs in mice injected with L-plastin peptides

The histological sections of the heart, liver, and pancreas were assessed by a pathologist blinded to the injection conditions. There were no changes in the organs in response to the injections (Fig. S2). Although nuclei of the liver appear to be hyperactive (indicated by wavy arrows in Fig. S2; panel D) in the inhibitory LPL peptide injected mice, no sign of inflammation has been observed in the organ.

### Micro-CT and histomorphometry analyses

**Fig. 2:**
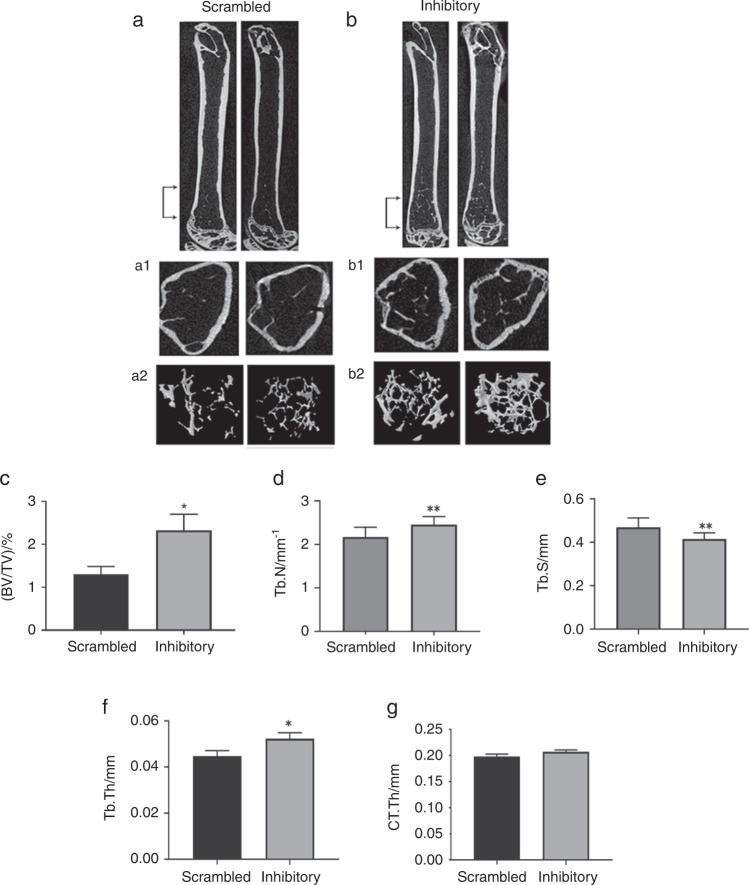
Representative longitudinal sections of femurs isolated from mice injected with indicated peptides are shown in duplicates. Micro-CT 3D-construction of the trabecular and cortical bone area indicated by bracketed arrows (region of interest, ROI) in (**a** & **b**). **a1** and **b1** Representative cross-sections of the femur in the metaphyseal area revealed cortical and trabecular bone. **a2** and **b2:** **c**–**g** Comparison of the indicated micro-CT parameters were done in mice injected with scrambled and inhibitory peptides. Bone volume to total volume (BV/TV), trabecular number (Tb. N), trabecular spacing (Tb.S), trabecular thickness (Tb. Th), and cortical bone thickness (CT.Th) were measured in the bones of nine mice and provided as bar graphs. The analysis was done thrice. Two times five mice and one time, nine mice were used for each group. The data shown are the results obtained from the experiment done with nine mice (*n* = 9 per group). Statistical analyses were done using a standard Student’s *t* test. Data shown are mean ± SEM; *P < 0.05; **P < 0.01; vs. scrambled peptide injected mice. Data are also offered as a scatterplot in Fig. S4

**Fig. 3:**
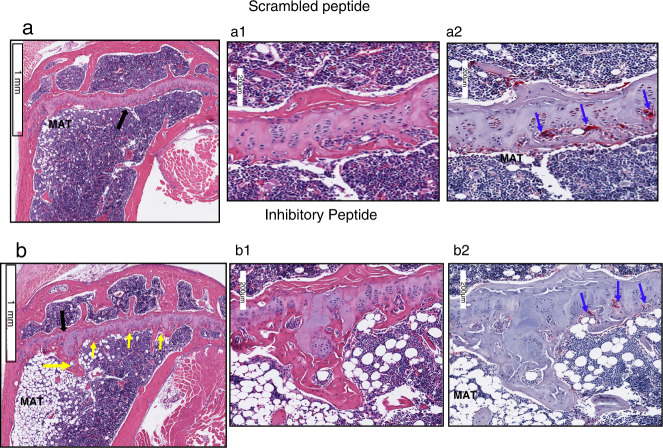
Bone sections were stained with H&E and TRAP-stains (*n* = 9 per group). H and E stained sagittal sections of the proximal tibia are shown (**a** and **b**). A black arrow in (**a** and **b**) points to the area of magnification shown in (**a1** and **b1**). The corresponding region of the magnified image is shown from the TRAP stained bone section (**a2** and **b2**). Scale Bar-1 mm in (**a** and **b**); 200 µm in (**a1**, **a2**, **b1**, and **b2**). Yellow arrows in (**b**) point to the trabecular bone, and blue arrows point to osteoclasts in TRAP-stained sections (**a2** and **b2**). MAT - marrow adipose tissue. Three bone sections from three different mice are shown for each injection in fig. S3

Femurs and tibias from mice injected with peptides were subjected to Micro-CT and histomorphometry analyses (Figs. 2 and 3, Fig. S3), respectively. Right femoral bones were analyzed in a Micro-CT unit (Fig. 3). Left femoral bones were also analyzed separately in a different Micro-CT unit (Fig. S4). Micro CT studies displayed a significant increase in BV/TV, trabecular number, and thickness in mice injected with the inhibitory LPL peptide (Fig. 2, panels b–e). As a result, a considerable decrease in trabecular separation was observed (Fig. 2, panels b2 and f; Fig. S4G). No significant change in the thickness of the cortical bone was observed between the mice injected with scrambled or inhibitory LPL peptide (Fig. 2, panels b and g; Fig. S4E and G). Both Micro-CT analyses with left and right femoral bones provided comparable results (Fig. 2 and S4).

A representative histological section of the tibial bone sections stained with H&E and TRAP-stainings is shown in Fig. 3. Three bone sections from three different mice are displayed for each injection in Fig. S3. Static histomorphometric measurements demonstrated a significant increase in trabecular bone number and thickness, which corresponded with a decrease in trabecular separation (Table 1; Fig. 3 and Fig. S3). The cortical thickness (width), number of osteoclasts, and osteoblasts remain the same in mice injected with either the inhibitory or scrambled LPL peptide (Table 1). Static histomorphometric measurements of the tibial bones provided similar results as Micro-CT analyses done with both right and left femoral bones (Figs. 2 and 3). In dynamic histomorphometry analyses, no significant difference was observed in the mean bone formation rate and mineral apposition rate between the experimental groups (Table 1). These data suggest that an increase in the number and thickness of trabecular bone in mice injected with inhibitory LPL peptide could be due to the inhibition of osteoclasts’ activity and not osteoblasts.

**Table 1:** H &E and TRAP stained bone sections were used for analyses in BioQuant-Osteoimage analysis software

Parameters	36 + 14 weeks scrambled	36 + weeks inhibitory
Static		
Cancellous bone/%	10.65 ± 3.3	14.36 ± 2.86
Trabecular number/mm^−1^	1.06 ± 0.23	2.25 ± 0.46*
Trabecular thickness/µm	7.8 ± 1.6	12.43 ± 2.3**
Trabecular separation/µm	420.8 ± 35.92	302.6 ± 15.9**
Osteoclast number	29.93 ± 10.87	25.5 ± 8.58
Osteoblast number	179 ± 35.47	214 ± 42.81
Cortical width/µm	158 ± 7.3	165.8 ± 6.83
Dynamic		
MAR/(μm·d^−1^)	0.38 ± 0.05	0.42 ± 0.09
Bone formation rate/(μm^2^·μm^−1^ per day)	0.30 ± 0.08	0.29 ± 0.03

Data shown are mean ± SEM of nine mice from one experiment (*n* = 9 per group)

*P < 0.05 and **P < 0.01 vs. scrambled peptide injected mice. The standard Student’s *t* test was used to assess *P* values. The results represent one of the three experiments performed

**Fig. 4:**
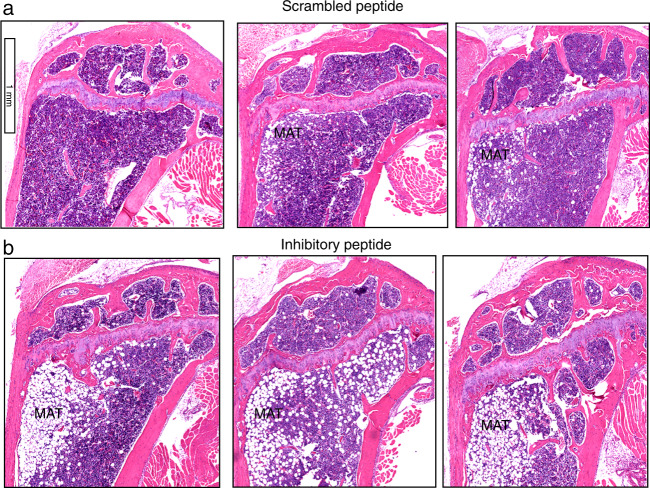
Data are shown in triplicates for each injection. Histological assessment of the proximal right tibial bone sections for adipose tissue in mice injected with scrambled (**a**) and inhibitory (**b**) peptide of Lplastin for 14 weeks. Data are shown in triplicates for each injection. Bone sections were stained with H&E, and marrow adipose tissue is denoted as MAT. *N* = 9 per group. Scale Bar-1 mm

Next, Histological sections of the tibial bones demonstrated an increase in bone marrow adiposity of the mice injected with the inhibitory peptide (Fig. 4b) compared with mice injected with scramble LPL peptide (Fig. 4a). This increase had no impact on the body weight of the mice (Fig. S1B). Therefore, these mice were not obese. Adipocytes and osteoblasts are derived from mesenchymal stem cells. One may think an increase in adipocyte differentiation may affect the differentiation of osteoblast and bone formation. As a result, a decrease in bone mass may occur. Here, we show no significant reduction in the number of osteoblasts in mice injected with the inhibitory peptide. However, an increase in the number and thickness of trabecular bone was observed (Table 1). Either the adipocyte or osteoblast formation is not occurring at the expense of each other. An increase in adipocyte formation is intriguing in mice injected with inhibitory peptide. Hence, further analyses are required to identify the mechanism by which adipocytes’ formation is augmented in these mice and the potential association of adipocytes with metabolic or energy requirements.

### Serum analyses

**Fig. 5:**
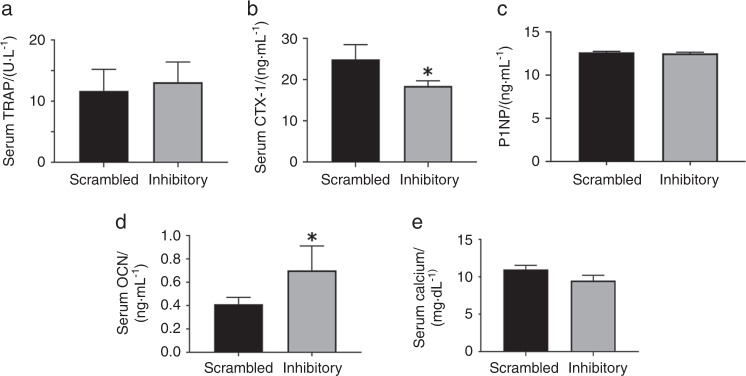
The respective ELISA kits were used to measure the serum levels of TRAP (**a**), CTX-1(**b**), P1NP (**c**), osteocalcin (OCN; **d**), and calcium (**e**). Data were assessed using the standard Student’s *t* test. The data represent the mean ± SEM of nine mice per group (*n* = 9 per group). *P < 0.05 vs. scrambled peptide injected mice. Analyses were done twice from two different experiments and obtained comparable results

Serum was analyzed for bone resorption (TRAP, CTX-1) and formation (P1NP, osteocalcin) markers by ELISA (Fig. 5). The levels of TRAP and calcium remains the same in mice injected with scrambled and inhibitory LPL peptides (Fig. 5, panels a and e). However, a decrease in the C-terminal telopeptide levels of type 1 collagen (CTX-1) suggests a reduction in osteoclast activity (Fig. 5b). Serum levels of P1NP and osteocalcin serve as a marker for bone formation. Here we showed that the levels of P1NP remain the same in mice injected with either scrambled or inhibitory LPL peptide (Fig. 5c). Contrariwise, a significant increase in the level of osteocalcin, was observed in the serum of mice injected with inhibitory LPL peptide (Fig. 5d). Dynamic histomorphometry analysis failed to show an increase in bone formation (Table 1). A significant increase in the bone formation marker osteocalcin may occur independently of osteoblasts. Although the function of bone marrow adipocytes is still in question, it has been shown by others that it may serve as energy reservoirs for the bones.^18^ Also, serum osteocalcin was suggested as a measure for adiposity.^19^ The present findings of increased adiposity and osteocalcin in mice injected with the inhibitory LPL peptide raise important questions for future studies. Is there any relationship between adiposity and osteocalcin levels (Fig. 4b) for glucose and fat metabolism? Further studies are underway to determine the relationship of adiposity to osteocalcin levels and energy metabolism.

### Mechanical properties of the femoral diaphysis

**Fig. 6:**
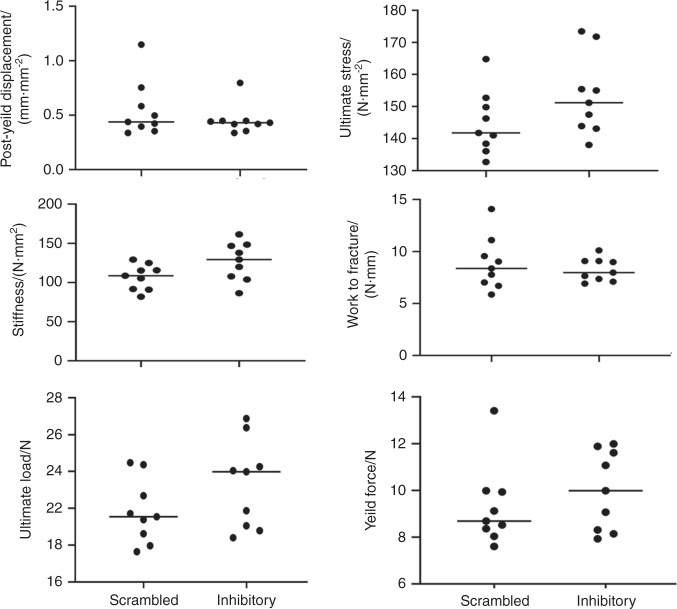
Data are given as scatter plots for the parameters, including post-yield displacement, ultimate stress, stiffness, work-to-fracture, ultimate load, and yield force. Statistical analyses were done using the Graph pad, and differences in the mice group have been analyzed using an unpaired *t*-test (two-tailed) with a significance value at <0.05. The data shown are the results obtained from the experiment done with nine mice/group

Micro-CT analyses demonstrated an increase in trabecular bone density in the metaphyseal region of the bone. However, there are no changes in cortical bone density (Figs. 2 and 3). Parameters analyzed to determine the bone’s material strength did not differ significantly between the groups used for the analyses (Fig. 6). No change in cortical bone density or thickness may be due to increased mineralization during growth, but little loss occurs during early aging (20). The predominant age-related bone loss ensues at the trabecular bone area at the early stage and continues throughout life in C57BL/6 mice. Bone loss is more in females than males.^16^ Our data provide evidence that an increase in trabecular density in mice injected with the inhibitory LPL peptide results from decreased osteoclast function. It has been suggested that the diaphyseal side of the bone exhibits periosteal expansion throughout life, while trabecular sites at proximal tibia or femur demonstrate age-related bone loss. Our data indicate that LPL is a novel target for trabecular bone loss. It is also believed that age-related changes may be evident at 18 months of age with further changes from 18–24 months.^20,21^ At the end of our studies, mice were 50 weeks old. The limitation here is that analyses have not been done in older age groups (ranging from ~72–96 weeks) to determine the potential impact of LPL peptide on cortical bone remodeling. Future studies will focus on this aspect.

## Discussion

Age-related bone loss causes a decrease in the number and thickness of trabecular or cancellous bone. Therefore, a reduction in connectivity and an increase in the fragility of bone occurs. In this study, we elucidated for the first time that injection of the small molecular weight inhibitory LPL peptide of LPL has the potential to suppress the function of osteoclasts without affecting the function of osteoblasts as compared with scrambled peptide injected mice. Our experiments’ most key outcome with the inhibitory LPL peptide is that it increases the trabecular bone density with no cortical bone density change. We have recently shown in LPL^-/-^ mice that LPL deficiency decreases cortical bone density via increasing the bone loss at the endosteal perimeter with no change in periosteal apposition.^14^ In the present study, we have observed no differences in cortical bone density between inhibitory and scrambled LPL peptide injected mice, suggesting that osteoclast-mediated bone resorption is regulated differently in the cortical region. LPL-dependent and -independent regulatory mechanisms occur at the bone’s metaphyseal and diaphyseal areas, respectively, in mice injected with the inhibitory LPL peptide. An increase in trabecular density in mice injected with the inhibitory LPL peptide validates this regulation. More studies are needed to characterize how does this specific phase of bone remodeling occurs.

While we found that osteoclast number was not reduced, but the inhibitory peptide of LPL repressed the activity reasonably, which is not too surprising. We have recently demonstrated that osteoclasts from L-plastin knockout (LPL^-/-^) mice are not defective in podosome formation. Therefore, they are capable of attaching to the bone surface. However, these osteoclasts failed to form NSZs and fully functional sealing rings in vitro, vital for bone resorption. Consistent with our observations of adequate action in the trabecular compartment,^14^ others have also shown that LPL acts primarily at trabecular sites to regulate bone resorption. LPL deficiency in mice resulted in a marked increase in trabecular bone density.^9^ Trabecular bone loss is critical to the pathology of osteoporosis. At the menopausal stage, an increase in women’s trabecular bone loss raises the chances for bone fragility.^22–24^ This bone loss reduced the number of trabecular bones and their connectivity in women and men.^25,26^ In C57BL/6 mice, age-related deterioration of trabecular bone occurs early and continues throughout life. This bone loss is more pronounced in females than in males.^16^ Mice models of aging-related bone loss is a beneficial model system because the bone loss in mice is comparable to that of humans.^16^ Bone turnover is higher in the cancellous bone area of aging mice and humans.^27,28^ Trabecular bone loss begins at about 3 months in C57BL/6 mice and disappears by 8–12 months in male and female mice. However, the extent of loss is more in female mice than males.^16,17^ As shown by us and others, trabecular bone was very minimal or not observed in mice injected with the scrambled peptide at 50 weeks. The inhibitory peptide of LPL makes osteoclasts dysfunctional and increased the trabecular density without affecting osteoblast function. We suggest this because no significant change in the bone formation rate and mineral apposition rate was observed between the groups tested.

Histomorphometry analyses demonstrated no changes in the number of osteoclasts and osteoblasts in mice injected with either scrambled or inhibitory LPL peptide. The bone formation was normal and unaffected in mice injected with the inhibitory LPL peptide. Dynamic histomorphometry analyses did not show an increase in bone formation in the trabecular region. Serum P1NP level corroborates this observation in mice injected with either scrambled or inhibitory LPL peptide. A part of the reason that the inhibitory LPL peptide did not affect bone formation is that osteoblasts do not express LPL. LPL is expressed in hematopoietically derived cells (e.g., leukocytes and osteoclasts) and abnormally in cancer cells.^29–32^ Our in vitro and in vivo experiments elucidated that LPL is critical for osteoclast function. Also, LPL is not required for osteoclastogenesis, osteoblastogenesis, and bone formation.^13^ L-plastin deficiency increases bone density via decreasing osteoclast function.^9,14^ Here we showed that inhibitory LPL peptide has an anti-catabolic, not anabolic effect. Several promising novel treatments have been developed to decrease osteoclasts’ function and thereby increase bone formation by osteoblasts.

Bisphosphonates, denosumab (a monoclonal antibody against RANKL), Romosozumab (anti-sclerostin monoclonal antibody), and odanacatib (a specific inhibitor to protease cathepsin K) are mostly used as anti-resorptive therapy.^25,33–37^ Denosumab reduced significantly vertebral, nonvertebral, and hip fractures compared with placebo and increase areal BMD compared with alendronate in phase 3 clinical studies.^33^ While denosumab and bisphosphonates have been shown to prevent skeletal-related events in patients with bone metastasis, there is a concern that it may cause atypical femoral fracture.^38–40^ Romosozumab or anti-sclerostin monoclonal antibody reduced the risk of vertebral fractures, and it increased the bone mineral density of the lumbar spine. A significant reduction in bone resorption markers, increases in bone formation markers, and improved BMD in postmenopausal women, reducing the risk of clinical fracture.^41–43^ However, data from phase 3 randomized controlled trials with this antibody suggested a possibility for cardiovascular risk.^44^ A decrease in bone resorption by the inhibitory LPL peptide, together with secondary bone formation due to coupling during bone remodeling, can maintain and stabilize bone mass. The inhibitory LPL peptide has the anti-resorptive property and could be developed as a prospective therapeutic agent to treat osteoporosis.

Calcein labeling of mice demonstrated no change in bone formation and mineral apposition rate. Also, no changes in the levels of P1NP, a bone formation marker, was observed in mice injected with either inhibitory or scrambled LPL peptide corroborate the experiments with calcein labeling. However, a significant increase in osteocalcin was observed. Serum osteocalcin is a valid marker to determine bone formation when bone formation and resorption are uncoupled.^45–47^ Osteocalcin is stored in the carboxylated form in the mineralized bone tissue. Due to acidic pH during bone resorption, decarboxylated osteocalcin is released as undercarboxylated osteocalcin from the bone matrix into the circulation.^48^ Since bone resorption is reduced in mice injected with the inhibitory LPL peptide, osteocalcin in the serum is not due to osteoclast activity. Osteocalcin was shown to have a broader role in other organs besides bone.^49,50^

Under the uncoupled bone remodeling (e.g., aging and pathological conditions), adipogenesis was suggested to be a default pathway if osteogenesis stimuli are absent.^51–53^ A decrease in bone mass was observed when there was an increase in marrow adipose tissue.^54^ The formation of marrow adipocytes does not seem to occur due to the suppression of osteoblast formation, while both are derived from mesenchymal stem cells. The equal number of osteoblasts and an increase in the thickness and the number of trabecular bone suggests that the increase is due to osteoclast activity inhibition. An increase in adipocytes at the trabecular or metaphyseal region also did not affect the activity of osteoblasts. Dynamic histomorphometry analysis confirmed no changes in the rate of bone formation in mice injected with the inhibitory peptide compared with mice injected with scrambled peptide. Especially during aging, bone cells require significant energy for active bone formation.^55,56^ Although more experiments are needed, an increase in serum osteocalcin and marrow adipose tissue near the growth plate area suggests that there may be an endocrine loop that exists between osteoblasts and adipose tissue. It seems that bone marrow cells can increase adiposity in response to the inhibitory LPL peptide injection. More experiments are needed to confirm the mechanism involved in adipose tissue formation.

The detection of improved bone quality in aging mice injected with the inhibitory LPL peptide justifies LPL as a novel therapeutic target. However, this study did not examine the molecular basis and physiologic consequence of increased marrow adiposity, nor did we explore the link between adiposity and osteocalcin expression. Therefore, future studies will explore the relationship between adiposity and osteoblast function. In addition, we will examine the impacts of LPL inhibitory peptides on other models that demonstrate bone loss, such as ovariectomized mice as an estrogen deficiency model, estrogen-dependent osteopenia, and disuse induced osteopenia models, which show osteoclast activation and bone loss.

## Conclusions

Several targeted therapies are available to prevent and treat osteoporosis by blocking osteoclast activity. Several peptides (e.g., growth factors and bone-related proteins) have also been shown to stimulate bone healing and bone regeneration^57^ ^(rev.^ ^58^^)^. Cell-penetrating peptides fused with a transcriptional factor, miRNA-29b, or BMP2, increased bone formation in a rabbit calvarial-defect model.^59–61^ Injection of TAT-fused NEMO binding domain peptide blocked osteoclastogenesis and bone erosion in inflammatory arthritis.^62^ This paper provides evidence that the peptide derived from an actin-bundling protein can prevent osteoclast function and increase trabecular bone density in aging mice. The inhibitory LPL peptide could be used as an anti-resorptive therapeutic peptide to reduce osteoclast-mediated bone loss and osteoporosis. Current therapies for osteoporosis include agents that target osteoclasts to reduce bone resorption. Therapeutics are required to inhibit osteoclast function without affecting the function of osteoblasts. Based on in vitro,^12–14^ and in vivo analyses, we believe LPL is emerging as a novel therapeutic target due to its cell-specific activation in cancer and hematopoietic cells and not in other normal cells. LPL inhibitory peptide did not affect the formation of osteoblasts or osteoclasts. It increases bone formation via decreasing osteoclast activity. Future studies will focus on the design and development of nanocarriers for the sustained release of peptides with higher efficiency and eliminate the injection of mice repeatedly.

## Materials and methods

### Materials

Rhodamine-phalloidin and all other chemicals were purchased from Sigma (St. Louis, MO). Mounting solutions for mounting of coverslips were purchased from Thomas Scientific (Swedesboro, NJ) or Vector Labs (Burlingame, CA). TAT-fused small molecular weight amino-terminal L-plastin (sNT-LPL) peptides [scrambled (TAT-SRGGMVEEAD) and inhibitory (TAT-MARGSVSSDEE) LPL peptides] were made from Genscript Co. (Piscataway (NJ).

### Transduction of TAT-fused small molecular weight peptides into osteoclasts in vitro and staining of osteoclasts with rhodamine-phalloidin

Osteoclasts were generated from RAW cells, as described.^12,63^ After osteoclasts were kept in the serum-free α-MEM medium for 2 h, scrambled and inhibitory LPL peptides were added to respective osteoclast cultures in the presence of TNF-α (20 ng·mL^−1^). After transduction for 15–30 min, cells were replated on dentine slices with individual peptide in the presence of TNF-α for 3–4 h and 12–14 h for actin staining to detect NSZs and sealing rings, respectively. Osteoclasts were stained with rhodamine-phalloidin to define actin distribution, as described previously.^64,65^ Actin stained osteoclasts were photographed using a 510 Meta laser scanning confocal microscope (Carl Zeiss). Images were stored in a TIF image format and processed by Adobe Photoshop (Adobe Systems Inc., Mountain View, CA).

### Dentine matrix resorption assay

After transduction for 15–30 min with the LPL peptides, osteoclasts were replated on dentine slices and incubated for 12–16 h in the presence of TNF-α (20 ng·mL^−1^) and respective peptide as described.^11,13^ All treatments were done in triplicates. Subsequently, dentin slices were washed with PBS and sonicated in 1 mol·L^−1^ NaOH for 1 min to remove cells. The slices were washed several times with water and stained with Meyer’s acid hematoxylin (Sigma, St. Louis, MO). Resorption pits were imaged under 40X objective in a Zeiss phase-contrast microscope fitted with a SPOT camera (Diagnostic Instruments, Alexandria, VA, USA). Images were stored in a TIF image format and processed by Adobe Photoshop (Adobe Systems Inc.). The resorption pit area was measured from the free-hand traced perimeter using the Scanalytics software (Scanalytics Inc., IP Lab, Fairfax, VA). About 25–30 pits/slice and three slices from each experiment were scanned. Statistical analysis was performed by Students *t* Test (INSTAT; version 6, Graph pad software, Graph Pad Inc., San Diego, CA).

### Injection of peptides into aging mice

Female C57BL/6 mice (age 35 weeks; mean body weight, 32 g) were bought from Charles River (Maryland, USA). Mice were kept in the facility for a week for acclimatization before injection with peptides. Mice were randomly divided into three groups (9 mice/group). Group 1 received saline (used as control). Groups 2 and 3 received injections of scrambled and inhibitory peptides of LPL, respectively. Groups 2 and 3 received doses of 100 μL of saline containing 50 μg peptide of interest (1.5 mg·kg^−1^). Saline or peptide of interest was administered subcutaneously for 14 weeks. At first, the injection was given every day (5 days per week) for 7 weeks, and later the injection was given on alternate days (3 days per week) for another 7 weeks, as indicated. Saline and control scrambled peptide injected mice demonstrated very similar results in the first set of experiments. Therefore, the subsequent two experiments were performed only with scrambled and inhibitory peptides of LPL. Results obtained from mice injected with control scrambled and inhibitory peptides of LPL are provided. Animal weights were taken every 2 weeks for up to 13 weeks. All animals were euthanized at 14 weeks; long bones, blood samples, and organs such as liver, kidney, and heart were collected for analyses.

### Micro-computed tomography (Micro CT) analysis

The femurs were dissected after the peptides’ injection, and three-dimensional *Micro CT* analysis was performed using a Skyscan 1172 system (Bruker, Kontich, Belgium) at 55 kV (167 µA) and a 10 µm voxel size, as described.^66,67^ The skeletal parameters were assessed by micro-CT and followed published nomenclature guidelines.^68^ Trabecular bone microarchitecture [trabecular bone volume fraction (BV/TV), trabecular bone thickness (Tb. Th), trabecular number (Tb. N), and trabecular separation (Tb. Sp)] was analyzed in a manually delineated region of interest 0.25–2.5 mm proximal to the distal femoral growth plate. Cortical thickness (Cs. Th) was 0.6 mm region at the femoral mid-diaphysis.

### Bone histomorphometry and histological analyses of soft organs

Bone Histomorphometry was done in long bones collected from mice as described.^64,69^ Long bones (tibia and femur) were fixed in phosphate-buffered 10% formalin and decalcified in 14% EDTA for 10–14 d. Bones were washed sequentially with 50%, 70%, and 90% ethanol and embedded. Longitudinal sections of 5 µm thickness were made and stained with TRAP and H & E staining. Mineral apposition and bone formation rates were measured in mice injected with calcein on days 2 and 7 before sacrificing. Bones were preserved in 90% ethanol, embedded, and longitudinal sections (5 µm thickness) were made.^69^ Static and dynamic histomorphometric parameters were analyzed using Bio-Quant software. All analyses were done distal to the growth plate region of the metaphysis. To estimate the bone formation rate, double- and single-labeled areas in tibias were traced and calculated as described.^70,71^ All parameters were measured and expressed as per the terminology recommended by the Histomorphometry Nomenclature Committee of the American Society for Bone and Mineral Research.^72^ Osteoclast numbers were counted in TRAP-stained bone sections, and osteoblasts were counted in H & E stained sections. Organs were fixed in 10% buffered formalin, and sections were stained with H & E stain. Histological sections (bone and other tissues) were scanned in the Aperio Scanscope CS system (Vista, CA). A pathologist blinded to the treatment conditions evaluated the sections for any disorder.

### Biomechanical testing of femoral bones from mice injected with peptides

Left femoral bones were also analyzed separately in a different Micro-CT unit, and then they were used for three-point bending tests. A three-point bending test was performed on femurs using the miniature bending apparatus as described.^73^ Femora (*n* = 9 per group) were mechanically tested to failure in three-point bending using Instron 8841 with a displacement support rate of 0.1 mm·s^−1^ and support span 7 mm apart. The failure occurred directly beneath the loading point, at the 50% length of the femur, a site comprised entirely of cortical bone. Force-displacement data were collected and analyzed to determine whole-bone (structural) mechanical properties (stiffness, ultimate force, post-yield displacement, and work-to-fracture). The mechanical properties were normalized for bone size, and ultimate strength and stress (N·mm^−2^) were calculated as described.^73,74^

### Enzyme-linked immunosorbent assay (ELISA)

Serum markers of bone resorption [tartrate-resistant acid phosphatase (TRAP), collagen C-terminal telopeptide (CTX-I)], and bone formation [osteocalcin, and procollagen1 N-terminal peptide (P1NP)] were measured in duplicates using respective ELISA Kits (Immunodiagnostics Systems, and LS-Bio Systems). The serum was separated from blood samples collected by cardiac puncture at the time of euthanizing the mice. Then the separated serum samples were frozen at −80 °C until used for the measurements. Also, calcium serum levels were measured using a calcium detection kit (Biovision, Inc., Milpitas, CA) according to the manufacturer’s instructions.

### Statistical analysis

Results are presented as mean ± SD or SEM with sample size (*n*) indicating the number of independent experiments. Statistical significance was assessed by Student’s *t* test or two-way ANOVA (Graph Pad Software, Graph Pad Inc, San Diego, CA). A probability value at *P* < 0.05 was considered statistically significant.

### Study approval

All animal procedures, including the injection of peptides of interest, were performed following appropriate guidelines and approval (approved protocol number; #417006) of the University of Maryland Institutional Animal Care and Use Committee (IACUC).

## Supplementary information

Supplementary Figures

## Patent for the peptide

United States Patent and Trade Mark Office Confirmation number: 3483; Publication No. US-2020-0255486-A1 and date 08/13/2020; Title: Compositions and methods for the inhibition of L-plastin activity in osteoclasts to reduce bone loss.
