# Supplementary material for: Peptidomimetic inhibitor of L-plastin reduces osteoclastic bone resorption in aging female mice
Source: Bone Res. 2021 Apr 9;9:22. doi: 10.1038/s41413-020-00135-9 (PMC8035201; doi:10.1038/s41413-020-00135-9)

**Fig. S1:** Measurement of changes in body weight and appearance of mice injected with indicated peptides for 13 weeks.

About nine aging female mice at the age of 36 weeks were injected with scrambled and inhibitory LPL peptide for 14 weeks. Mice were photographed on week 13^th^ (A). Mice were weighed every two weeks until 13 weeks. Bodyweight is provided as a bar graph in B, and two-way ANOVA was used to compare the body weight in nine mice (n=9 per group). Statistically, the two groups did not differ in weight at indicated weeks of measurement (mean± SEM).


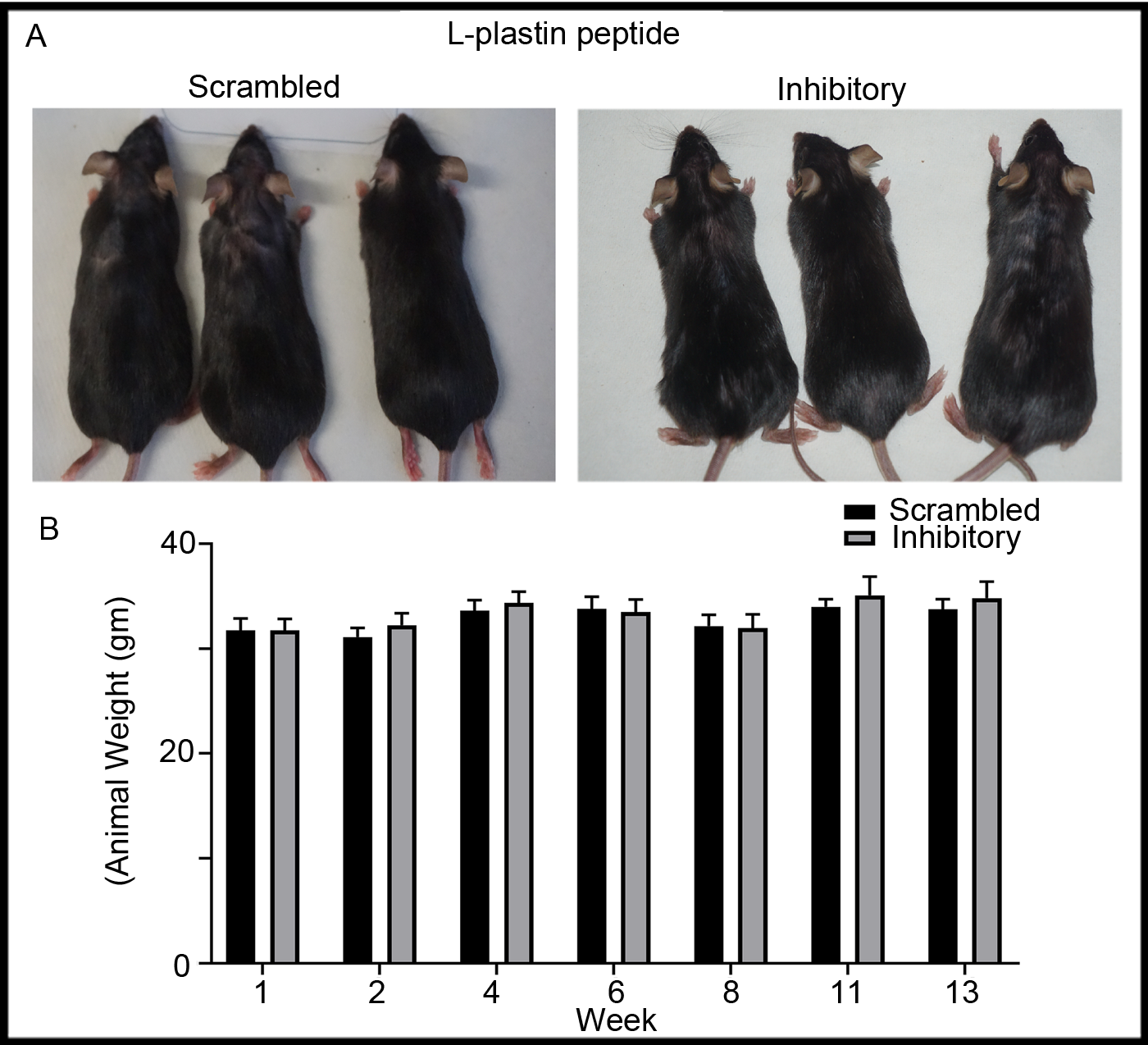


**Supplementary Figures**

**Fig. S2:** Histological analyses in different organs of mice injected with L-plastin peptides

Histological sections of the organs (kidney, heart, and liver) isolated from mice injected with scrambled (A) or inhibitory LPL peptides (B) of LPL is shown. Organs were isolated from five mice (n=5/group) of each injection and analyzed for any changes or inflammation. A representative histological section of the soft organ from each injection is shown. An arrow in the kidney, heart, and liver sections in A and B points to the area of magnification shown at the right panel. An asterisk in the liver panels of A and B indicates the area of magnification shown in C and D. Wavy arrows in D point to the nucleus. Scale bar: A and B; left panels - 2mm; right panels- 200µm; C and D-50µm.


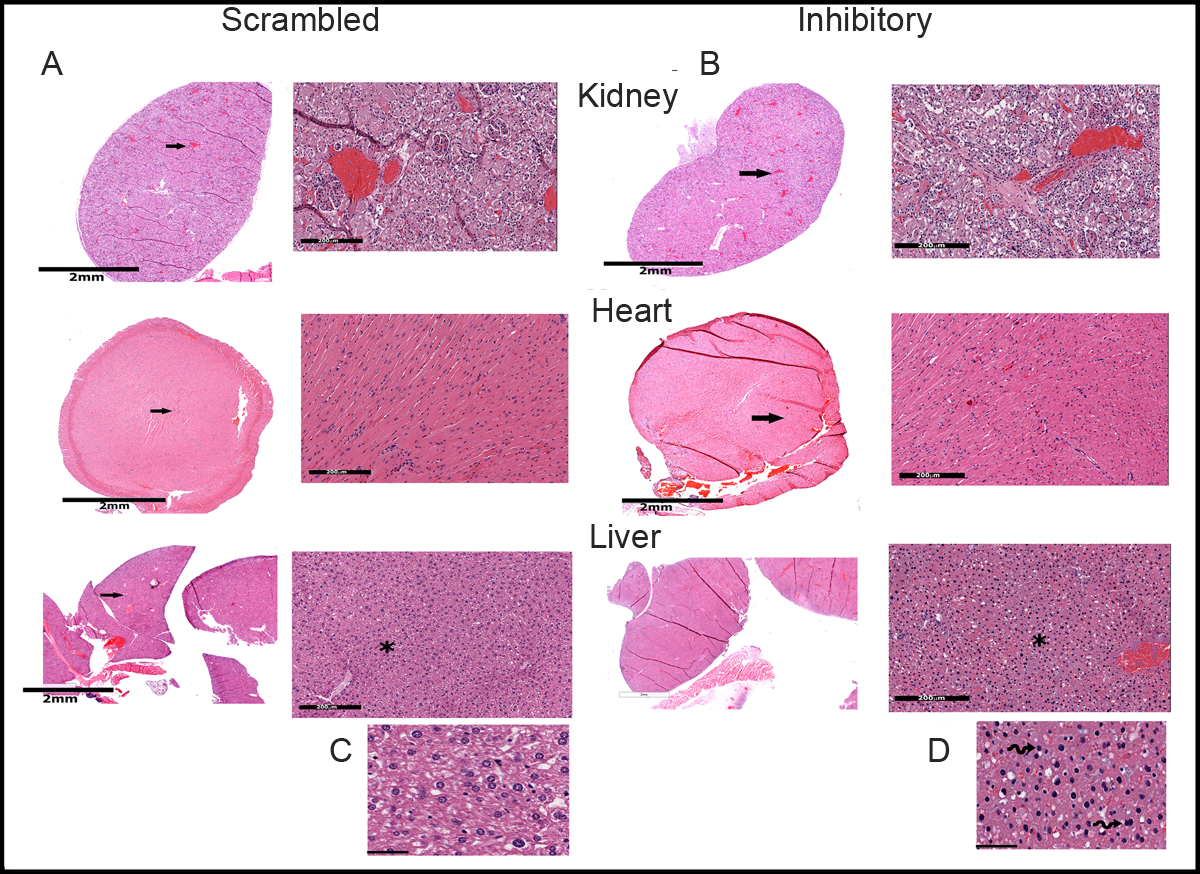


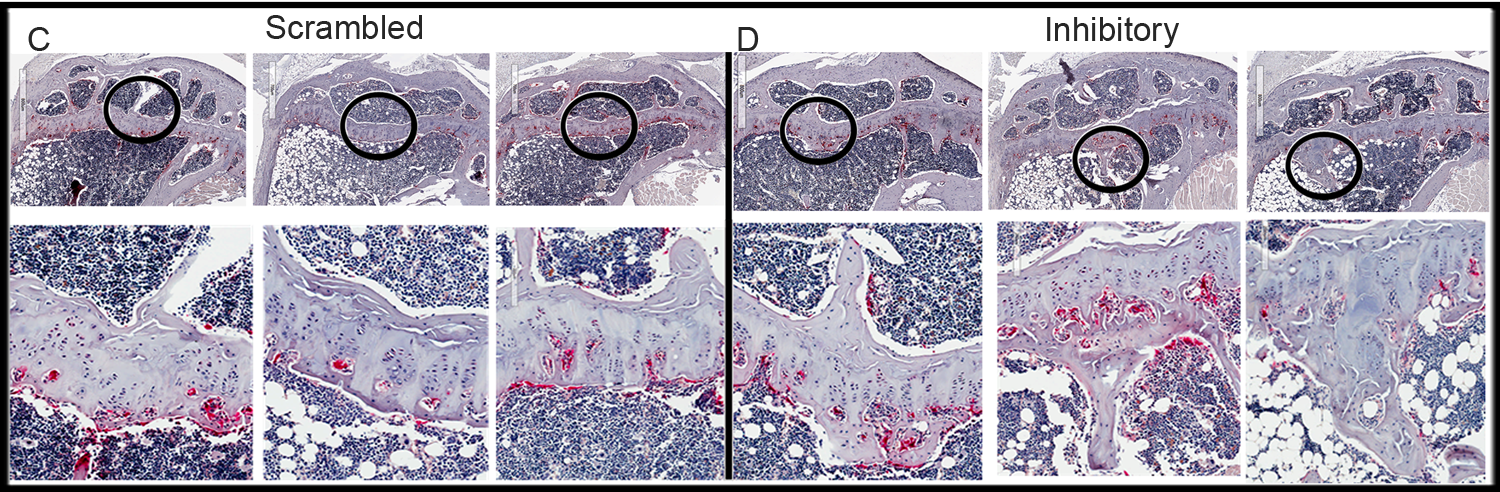


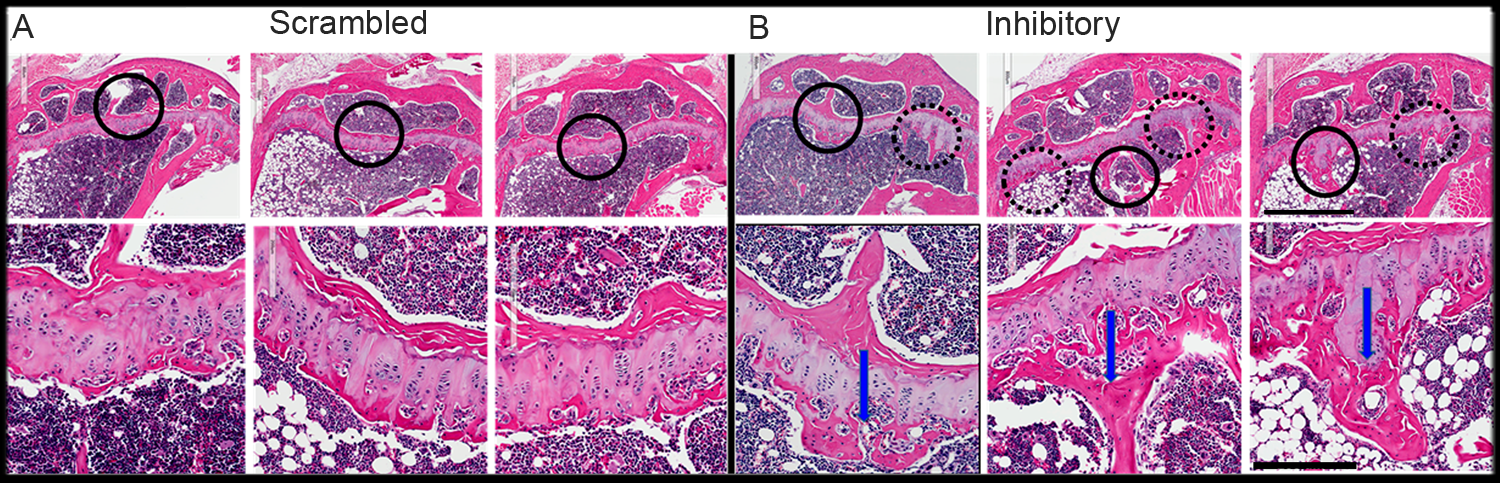


**Fig. S3**: Histological analysis of the proximal tibial bone sections of mice injected with scrambled (A and C) and inhibitory (B and D) LPL peptides as well as PBS (E and F) for 14 weeks.

Bone sections were stained with H&E (A, B, and E) and TRAP-stains (C, D, and F). Three bone sections from three different mice are shown for each injection. Black circles represent the area of magnifications, and magnified images are shown in the corresponding bottom panels. Broken circles in panel B point to the area with more trabecular bone in mice injected with the inhibitory LPL peptide. Blue arrows point to the magnified trabecular bone in LPL peptide injected mice (B). Scale Bar- 700-800µm in A -F; 200µm in the bottom panels of A-D; 300µm in the bottom panels of E and F. The results represent one of the three experiments performed with similar results.

**Reviewer 1; comment 1:** include data of negative control (mice with saline injection)…”

**Response:** As suggested histological sections of PBS injected mice bones are provided. Thanks

**Fig. S4:**  Comparison of the micro-CT parameters in mice injected with scrambled and inhibitory LPL peptides**.**

**A-E:** Data are provided as scatterplots for the parameters provided as bar graphs in Figure 4. Bone parameters measured in the femoral bones of nine mice are provided as dot blots. Measurements are provided for bone volume to total volume (BV/TV), trabecular number (Tb.N), trabecular spacing (Tb.S), trabecular thickness (Tb.Th), and cortical bone thickness (CT.Th). Statistical analyses were done using the standard Student’s t-test. Data shown are mean ± SEM; *p<0.05; **p<0.01; vs. scrambled peptide injected mice. **F and G:** Micro-CT with 3D construction of the metaphyseal region. The transverse plane of the metaphyseal region 1.5mm proximal to the growth plate is shown. The figure shown is the representative of 9 mice/group.


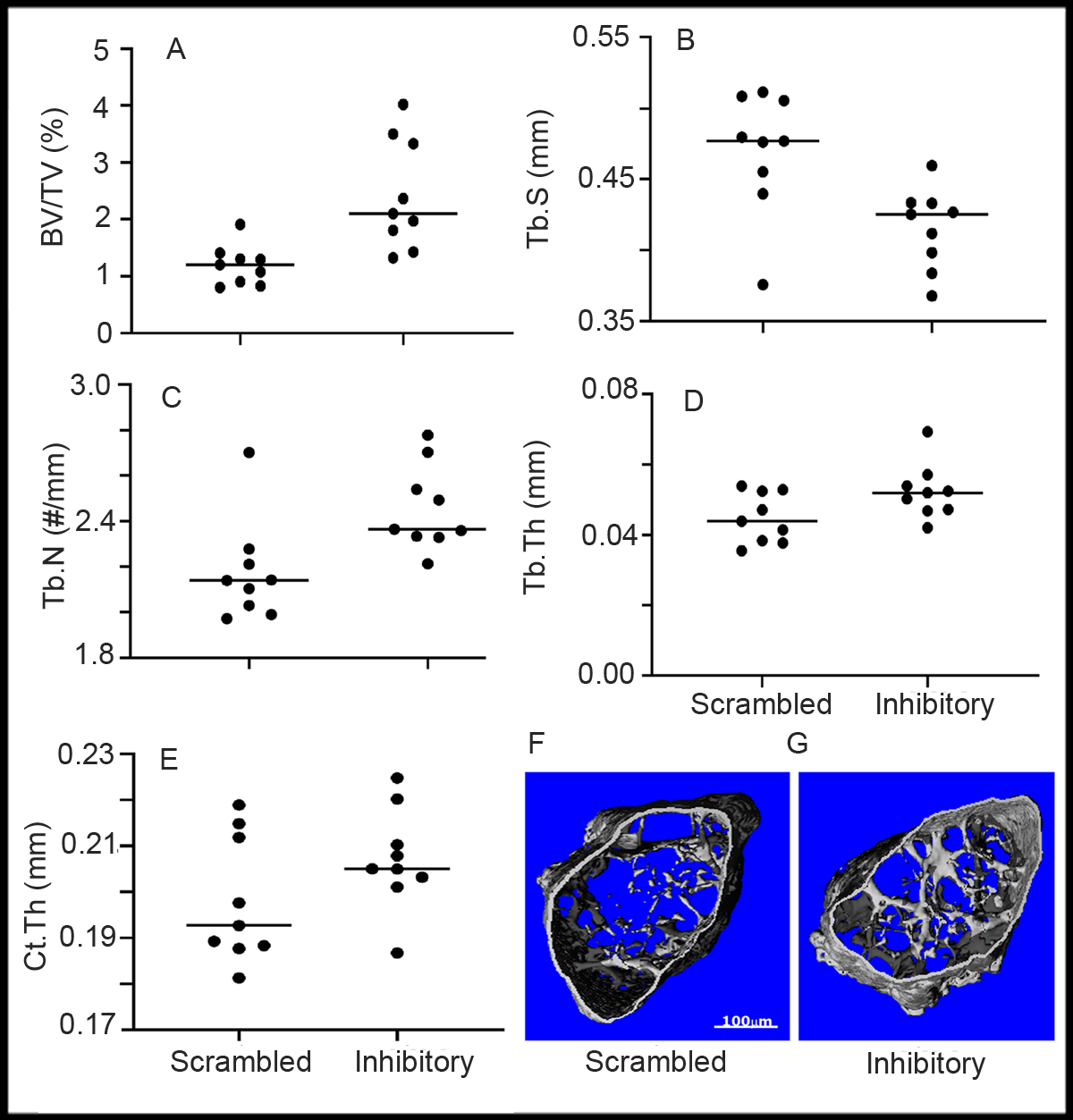

Supplement: Supplementary file 1 — Supplementary Figures [file 41413_2020_135_MOESM1_ESM.docx]
